# Bis(3,4-dimethoxy­benzoato-κ^2^ *O*,*O*′)(1,10-phenanthroline-κ^2^ *N*,*N*′)copper(II)

**DOI:** 10.1107/S1600536809052234

**Published:** 2009-12-09

**Authors:** Yaru Liu, Junshan Sun, Xiaoli Niu

**Affiliations:** a School of Science, North University of China, 030051 Taiyuan, Shanxi, People’s Republic of China; b Department of Materials and Chemical Engineering, Taishan University, 271021 Tai’an, Shandong, People’s Republic of China; c College of Foreign Languages, Shandong Agricultural University, 271000 Tai’an, Shandong, People’s Republic of China

## Abstract

The asymmetric unit of the title compound, [Cu(C_9_H_9_O_4_)_2_(C_12_H_8_N_2_)], contains one half-mol­ecule, the complete mol­ecule being generated by a twofold rotation axis. The Cu^II^ atom exhibits a six-coordinated distorted octa­hedral geometry with two N atoms from the phenanthroline ligand [Cu—N 2.007 (2) Å] and four O atoms from two 3,4-dimethoxy­benzoate ligands [Cu—O 1.950 (1) and 2.524 (1) Å]. The difference in Cu—O bond distances indicates a strong Jahn–Teller effect. In the crystal, C—H⋯π inter­actions result in chains of mol­ecules along the *c* axis.

## Related literature

For metal–1,10-phenanthroline complexes with unusual features, see: Ma *et al.* (2004 ▶); Bi *et al.* (2004 ▶).
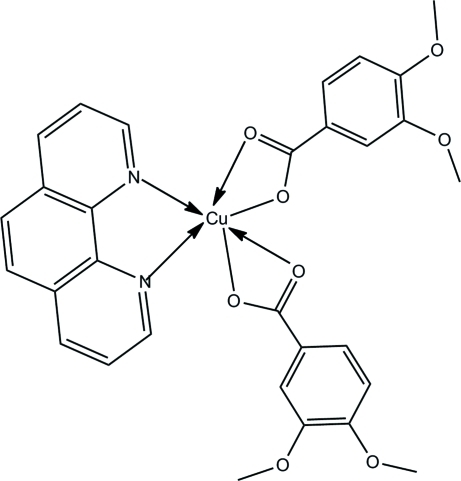


## Experimental

### 

#### Crystal data

[Cu(C_9_H_9_O_4_)_2_(C_12_H_8_N_2_)]
*M* *_r_* = 606.07
Monoclinic, 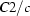

*a* = 12.1639 (10) Å
*b* = 11.4296 (9) Å
*c* = 19.7470 (16) Å
β = 104.027 (1)°
*V* = 2663.5 (4) Å^3^
*Z* = 4
Mo *K*α radiation
μ = 0.88 mm^−1^
*T* = 273 K
0.23 × 0.21 × 0.19 mm

#### Data collection

Bruker SMART APEX diffractometer
Absorption correction: multi-scan (*SADABS*; Bruker, 2005 ▶) *T*_min_ = 0.824, *T*_max_ = 0.851
6857 measured reflections
2351 independent reflections
2136 reflections with *I* > 2σ(*I*)
*R*_int_ = 0.059

#### Refinement

*R*[*F* ^2^ > 2σ(*F* ^2^)] = 0.030
*wR*(*F* ^2^) = 0.086
*S* = 1.01
2351 reflections
187 parameters
H-atom parameters constrained
Δρ_max_ = 0.30 e Å^−3^
Δρ_min_ = −0.38 e Å^−3^

### 

Data collection: *SMART* (Bruker, 2005 ▶); cell refinement: *SAINT* (Bruker, 2005 ▶); data reduction: *SAINT*; program(s) used to solve structure: *SHELXS97* (Sheldrick, 2008 ▶); program(s) used to refine structure: *SHELXL97* (Sheldrick, 2008 ▶); molecular graphics: *XP* in *SHELXTL* (Sheldrick, 2008 ▶); software used to prepare material for publication: *SHELXL97*.

## Supplementary Material

Crystal structure: contains datablocks I, global. DOI: 10.1107/S1600536809052234/ez2194sup1.cif

Structure factors: contains datablocks I. DOI: 10.1107/S1600536809052234/ez2194Isup2.hkl

Additional supplementary materials:  crystallographic information; 3D view; checkCIF report

## Figures and Tables

**Table 1:** Hydrogen-bond geometry (Å, °)

*D*—H⋯*A*	*D*—H	H⋯*A*	*D*⋯*A*	*D*—H⋯*A*
C8—H8*B*⋯*Cg*1	0.96	2.98	3.642 (3)	127

C1 is the centroid of the C22,C23,C29,C22′,C23′,C29′ ring.

**Fig. 1.**
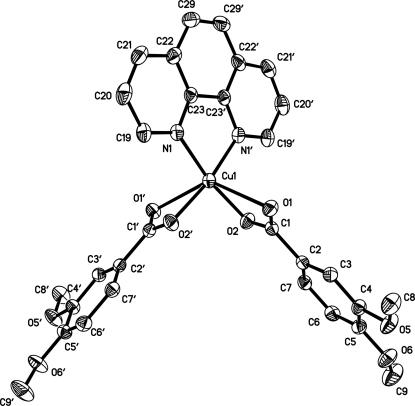
The molecular structure of the title compound, with atom labels and 30% probability displacement ellipsoids for non-H atoms. (Symmetry code: -x, y, 0.5 - z)

## supplementary crystallographic information

### Comment

Metal complexes with carboxylate ligands are among the most investigated complexes in the field of coordination chemistry. In addition, metal–1,10-phenanthroline complexes and their derivatives have attracted much attention during recent decades because of their unusual features (Ma *et al.*, 2004; Bi *et al.*, 2004). In this work, the title compound was obtained from the reaction of 3,4-dimethoxybenzoic acid and cupric acetate in the presence of 1,10-phenanthroline.

The molecular structure of the title complex is shown in Fig. 1. The Cu(II) atom exhibits a six-coordinated distorted octahedral geometry with two N atoms [Cu—N 2.007 (2) Å] from the phenanthroline ligand and four O atoms from the two 3,4-dimethoxybenzoate ligands [Cu—O 1.950 (1), 2.524 (1) Å]. The difference in Cu—O bond distances [Cu—O 1.950 (1), 2.524 (1) Å] indicates a strong Jahn-Teller effect. Two O atoms and two N atoms occupy the equatorial planar position with a slight deviation from the ideal plane of 0.0263 (2)Å, while two O atoms lie in the apical positions with an axis angle of 127.6 (2)° showing a large deviation from the normal 180°. A C8—H8b···π interaction results in chains of molecules along the c-axis [H8b···CG1 2.979 (3) Å, where CG1 is the centroid of the C22, C23, C29, C22^i^, C23^i^, C29^i^ ring; symmetry operator, i: -x, y, 0.5 - z].

### Experimental

The reaction was carried out by the solvothermal method. 3,4-dimythoxybenzoic acid (0.121 g, 2 mmol), cupric acetate (0.199 g, 1 mmol) and 1,10-phenanthroline (0.180 g, 1 mmol) were added to the airtight vessel with a 1:2 ratio of ethanol to water. The resulting blue solution was filtered. The filtrate was left for several days at room temperature to yield blue, block-shaped crystals.

The yield was 78% and elemental analysis: calc. for C_30_H_26_CuN_2_O_8_: C 59.45, H 4.32, N 4.62; found: C 59.31, H 4.49, N 4.53. The elemental analyses were performed with a PERKIN ELMER MODEL 2400 SERIES II.

### Refinement

The *U*_iso_(H) values were set at 1.2*U*_eq_(C—H) for the H atoms in the phenanthroline and aromatic ring, and 1.5*U*_eq_(C—H) for the methyl moiety. As the diffraction intensities were of high quality, the The H atoms could be located in difference Fourier maps.

### Crystal data

**Table:**

[Cu(C_9_H_9_O_4_)_2_(C_12_H_8_N_2_)]	*F*(000) = 1252
*M**_r_* = 606.07	*D*_x_ = 1.511 Mg m^−^^3^
Monoclinic, *C*2/*c*	Mo *K*α radiation, λ = 0.71073 Å
*a* = 12.1639 (10) Å	Cell parameters from 4483 reflections
*b* = 11.4296 (9) Å	θ = 2.5–28.3°
*c* = 19.7470 (16) Å	µ = 0.88 mm^−^^1^
β = 104.027 (1)°	*T* = 273 K
*V* = 2663.5 (4) Å^3^	Block, blue
*Z* = 4	0.23 × 0.21 × 0.19 mm

### Data collection

**Table:**

Bruker SMART APEX diffractometer	2351 independent reflections
Radiation source: fine-focus sealed tube	2136 reflections with *I* > 2σ(*I*)
graphite	*R*_int_ = 0.059
φ and ω scans	θ_max_ = 25.1°, θ_min_ = 2.1°
Absorption correction: multi-scan (*SADABS*; Bruker, 2005)	*h* = −14→14
*T*_min_ = 0.824, *T*_max_ = 0.851	*k* = −10→13
6857 measured reflections	*l* = −23→22

### Refinement

**Table:**

Refinement on *F*^2^	Primary atom site location: structure-invariant direct methods
Least-squares matrix: full	Secondary atom site location: difference Fourier map
*R*[*F*^2^ > 2σ(*F*^2^)] = 0.030	Hydrogen site location: inferred from neighbouring sites
*wR*(*F*^2^) = 0.086	H-atom parameters constrained
*S* = 1.00	*w* = 1/[σ^2^(*F*_o_^2^) + (0.045*P*)^2^ + 1.2527*P*] where *P* = (*F*_o_^2^ + 2*F*_c_^2^)/3
2351 reflections	(Δ/σ)_max_ = 0.001
187 parameters	Δρ_max_ = 0.30 e Å^−^^3^
0 restraints	Δρ_min_ = −0.37 e Å^−^^3^

### Special details

**Table:**

Geometry. All e.s.d.'s (except the e.s.d. in the dihedral angle between two l.s. planes) are estimated using the full covariance matrix. The cell e.s.d.'s are taken into account individually in the estimation of e.s.d.'s in distances, angles and torsion angles; correlations between e.s.d.'s in cell parameters are only used when they are defined by crystal symmetry. An approximate (isotropic) treatment of cell e.s.d.'s is used for estimating e.s.d.'s involving l.s. planes.
Refinement. Refinement of *F*^2^ against ALL reflections. The weighted *R*-factor *wR* and goodness of fit *S* are based on *F*^2^, conventional *R*-factors *R* are based on *F*, with *F* set to zero for negative *F*^2^. The threshold expression of *F*^2^ > σ(*F*^2^) is used only for calculating *R*-factors(gt) *etc*. and is not relevant to the choice of reflections for refinement. *R*-factors based on *F*^2^ are statistically about twice as large as those based on *F*, and *R*- factors based on ALL data will be even larger.

### Fractional atomic coordinates and isotropic or equivalent isotropic displacement parameters (Å^2^)

**Table:**

	*x*	*y*	*z*	*U*_iso_*/*U*_eq_	
Cu1	0.0000	0.96118 (3)	0.2500	0.03588 (14)	
O1	0.00052 (11)	0.87873 (12)	0.36863 (7)	0.0437 (3)	
O2	0.10961 (12)	0.84452 (12)	0.29637 (7)	0.0445 (3)	
O5	0.11674 (13)	0.56453 (14)	0.55593 (8)	0.0539 (4)	
O6	0.27611 (14)	0.44980 (13)	0.52542 (9)	0.0592 (4)	
N1	−0.10829 (13)	1.09400 (15)	0.21869 (8)	0.0389 (4)	
C1	0.07772 (15)	0.82281 (16)	0.35206 (9)	0.0364 (4)	
C2	0.13570 (15)	0.72454 (16)	0.39696 (9)	0.0341 (4)	
C3	0.09898 (15)	0.69411 (16)	0.45613 (10)	0.0365 (4)	
H3	0.0407	0.7364	0.4676	0.044*	
C4	0.14771 (16)	0.60242 (17)	0.49769 (10)	0.0385 (4)	
C5	0.23698 (17)	0.53957 (16)	0.48098 (11)	0.0406 (5)	
C6	0.27502 (16)	0.57054 (18)	0.42332 (11)	0.0418 (5)	
H6	0.3349	0.5300	0.4126	0.050*	
C7	0.22402 (16)	0.66243 (17)	0.38092 (10)	0.0389 (4)	
H7	0.2493	0.6823	0.3416	0.047*	
C8	0.0266 (2)	0.6253 (2)	0.57448 (12)	0.0583 (6)	
H8A	0.0494	0.7045	0.5868	0.087*	
H8B	0.0079	0.5872	0.6135	0.087*	
H8C	−0.0385	0.6255	0.5356	0.087*	
C9	0.3730 (2)	0.3888 (3)	0.51835 (16)	0.0801 (9)	
H9A	0.3578	0.3514	0.4735	0.120*	
H9B	0.3926	0.3307	0.5544	0.120*	
H9C	0.4349	0.4426	0.5223	0.120*	
C19	−0.21817 (17)	1.0896 (2)	0.18725 (11)	0.0482 (5)	
H19	−0.2525	1.0172	0.1756	0.058*	
C20	−0.28296 (19)	1.1910 (2)	0.17132 (12)	0.0588 (6)	
H20	−0.3597	1.1854	0.1497	0.071*	
C21	−0.23515 (19)	1.2974 (2)	0.18712 (11)	0.0547 (6)	
H21	−0.2793	1.3646	0.1774	0.066*	
C22	−0.11811 (18)	1.30632 (18)	0.21845 (10)	0.0454 (5)	
C23	−0.05979 (16)	1.20062 (17)	0.23347 (9)	0.0375 (4)	
C29	−0.0562 (2)	1.41293 (19)	0.23526 (11)	0.0541 (6)	
H29	−0.0942	1.4839	0.2257	0.065*	

### Atomic displacement parameters (Å^2^)

**Table:**

	*U*^11^	*U*^22^	*U*^33^	*U*^12^	*U*^13^	*U*^23^
Cu1	0.0365 (2)	0.0395 (2)	0.0311 (2)	0.000	0.00731 (14)	0.000
O1	0.0448 (7)	0.0459 (8)	0.0407 (7)	0.0081 (6)	0.0110 (6)	0.0047 (6)
O2	0.0497 (8)	0.0496 (8)	0.0357 (7)	0.0074 (6)	0.0134 (6)	0.0075 (6)
O5	0.0605 (9)	0.0624 (9)	0.0452 (8)	0.0236 (7)	0.0253 (7)	0.0197 (7)
O6	0.0618 (10)	0.0582 (10)	0.0624 (10)	0.0301 (8)	0.0244 (8)	0.0214 (8)
N1	0.0365 (8)	0.0467 (9)	0.0323 (8)	0.0016 (7)	0.0060 (7)	−0.0015 (7)
C1	0.0377 (9)	0.0363 (10)	0.0330 (9)	−0.0037 (8)	0.0044 (8)	−0.0019 (8)
C2	0.0369 (9)	0.0339 (9)	0.0304 (9)	−0.0024 (8)	0.0062 (7)	−0.0038 (8)
C3	0.0359 (9)	0.0377 (10)	0.0362 (10)	0.0059 (8)	0.0093 (8)	−0.0022 (8)
C4	0.0400 (10)	0.0423 (11)	0.0345 (10)	0.0049 (8)	0.0115 (8)	0.0023 (8)
C5	0.0415 (10)	0.0381 (11)	0.0410 (11)	0.0074 (8)	0.0077 (9)	0.0009 (8)
C6	0.0391 (10)	0.0430 (10)	0.0449 (11)	0.0061 (8)	0.0133 (9)	−0.0075 (9)
C7	0.0426 (10)	0.0421 (10)	0.0336 (9)	−0.0013 (8)	0.0125 (8)	−0.0040 (8)
C8	0.0647 (14)	0.0700 (15)	0.0497 (12)	0.0195 (12)	0.0321 (11)	0.0105 (11)
C9	0.0833 (19)	0.0836 (19)	0.0792 (18)	0.0514 (16)	0.0313 (15)	0.0234 (15)
C19	0.0376 (10)	0.0640 (13)	0.0404 (11)	0.0020 (10)	0.0042 (9)	−0.0029 (10)
C20	0.0405 (11)	0.0834 (18)	0.0493 (13)	0.0151 (12)	0.0046 (10)	0.0050 (12)
C21	0.0580 (13)	0.0639 (15)	0.0431 (12)	0.0226 (12)	0.0139 (10)	0.0087 (11)
C22	0.0599 (12)	0.0508 (12)	0.0290 (9)	0.0124 (10)	0.0173 (9)	0.0053 (9)
C23	0.0435 (10)	0.0451 (11)	0.0255 (9)	0.0019 (8)	0.0116 (8)	0.0001 (8)
C29	0.0834 (15)	0.0426 (11)	0.0415 (12)	0.0092 (11)	0.0251 (11)	0.0036 (10)

### Geometric parameters (Å, °)

**Table:**

Cu1—O2^i^	1.950 (1)	C6—C7	1.392 (3)
Cu1—O2	1.950 (1)	C6—H6	0.9300
Cu1—N1^i^	2.007 (2)	C7—H7	0.9300
Cu1—N1	2.007 (2)	C8—H8A	0.9600
Cu1—O1	2.524 (1)	C8—H8B	0.9600
O1—C1	1.244 (2)	C8—H8C	0.9600
O2—C1	1.276 (2)	C9—H9A	0.9600
O5—C4	1.365 (2)	C9—H9B	0.9600
O5—C8	1.419 (2)	C9—H9C	0.9600
O6—C5	1.359 (2)	C19—C20	1.394 (3)
O6—C9	1.406 (3)	C19—H19	0.9300
N1—C19	1.331 (2)	C20—C21	1.351 (3)
N1—C23	1.355 (2)	C20—H20	0.9300
C1—C2	1.497 (3)	C21—C22	1.412 (3)
C2—C7	1.387 (3)	C21—H21	0.9300
C2—C3	1.392 (3)	C22—C23	1.396 (3)
C3—C4	1.374 (3)	C22—C29	1.429 (3)
C3—H3	0.9300	C23—C23^i^	1.443 (4)
C4—C5	1.406 (3)	C29—C29^i^	1.350 (5)
C5—C6	1.375 (3)	C29—H29	0.9300
			
O2^i^—Cu1—O2	93.72 (9)	C7—C6—H6	119.9
O2^i^—Cu1—N1^i^	170.07 (6)	C2—C7—C6	120.39 (18)
O2—Cu1—N1^i^	92.84 (6)	C2—C7—H7	119.8
O2^i^—Cu1—N1	92.84 (6)	C6—C7—H7	119.8
O2—Cu1—N1	170.07 (6)	O5—C8—H8A	109.5
N1^i^—Cu1—N1	81.69 (9)	O5—C8—H8B	109.5
O2^i^—Cu1—O1	91.61 (5)	H8A—C8—H8B	109.5
O2—Cu1—O1	57.40 (5)	O5—C8—H8C	109.5
N1^i^—Cu1—O1	98.20 (5)	H8A—C8—H8C	109.5
N1—Cu1—O1	114.98 (5)	H8B—C8—H8C	109.5
C1—O1—Cu1	77.28 (11)	O6—C9—H9A	109.5
C1—O2—Cu1	102.80 (12)	O6—C9—H9B	109.5
C4—O5—C8	116.78 (15)	H9A—C9—H9B	109.5
C5—O6—C9	118.78 (19)	O6—C9—H9C	109.5
C19—N1—C23	118.06 (18)	H9A—C9—H9C	109.5
C19—N1—Cu1	128.66 (15)	H9B—C9—H9C	109.5
C23—N1—Cu1	113.28 (12)	N1—C19—C20	121.5 (2)
O1—C1—O2	122.24 (17)	N1—C19—H19	119.3
O1—C1—C2	120.47 (17)	C20—C19—H19	119.3
O2—C1—C2	117.28 (16)	C21—C20—C19	120.5 (2)
C7—C2—C3	119.18 (17)	C21—C20—H20	119.7
C7—C2—C1	121.90 (17)	C19—C20—H20	119.7
C3—C2—C1	118.91 (16)	C20—C21—C22	119.9 (2)
C4—C3—C2	120.81 (17)	C20—C21—H21	120.1
C4—C3—H3	119.6	C22—C21—H21	120.1
C2—C3—H3	119.6	C23—C22—C21	115.9 (2)
O5—C4—C3	125.33 (17)	C23—C22—C29	118.47 (19)
O5—C4—C5	114.98 (16)	C21—C22—C29	125.62 (19)
C3—C4—C5	119.69 (18)	N1—C23—C22	124.09 (17)
O6—C5—C6	126.41 (18)	N1—C23—C23^i^	115.86 (10)
O6—C5—C4	113.81 (18)	C22—C23—C23^i^	120.05 (12)
C6—C5—C4	119.77 (18)	C29^i^—C29—C22	121.46 (12)
C5—C6—C7	120.13 (18)	C29^i^—C29—H29	119.3
C5—C6—H6	119.9	C22—C29—H29	119.3

Symmetry codes: (i) −*x*, *y*, −*z*+1/2.

### Hydrogen-bond geometry (Å, °)

**Table:**

C1 is the centroid of the C22,C23,C29,C22',C23',C29' ring.

**Table:**

*D*—H···*A*	*D*—H	H···*A*	*D*···*A*	*D*—H···*A*
C8—H8B···Cg1	0.96	2.98	3.642 (3)	127
